# Supplementary material for: Somatostatin expressing GABAergic interneurons in the medial entorhinal cortex preferentially inhibit layerIII-V pyramidal cells
Source: Commun Biol. 2020 Dec 10;3:754. doi: 10.1038/s42003-020-01496-x (PMC7728756; doi:10.1038/s42003-020-01496-x)
Supplement: Supplementary file 2 — Supplementary Information [file 42003_2020_1496_MOESM2_ESM.pdf]

Supplementary information

Somatostatin expressing GABAergic interneurons in the medial entorhinal cortex preferentially inhibit layerIII-V pyramidal cells

Miklós Kecskés[1]*, Nóra Henn-Mike[1]*, Ágnes Agócs-Laboda[1]*, Szilárd Szőcs[1], Zoltán Petykó[1], Csaba Varga[1]

[1]Szentágothai Research Center, Department of Physiology, Medical School, University of Pécs, H-7624 Pécs, Hungary

*Contributed equally to this work

Supplementary information

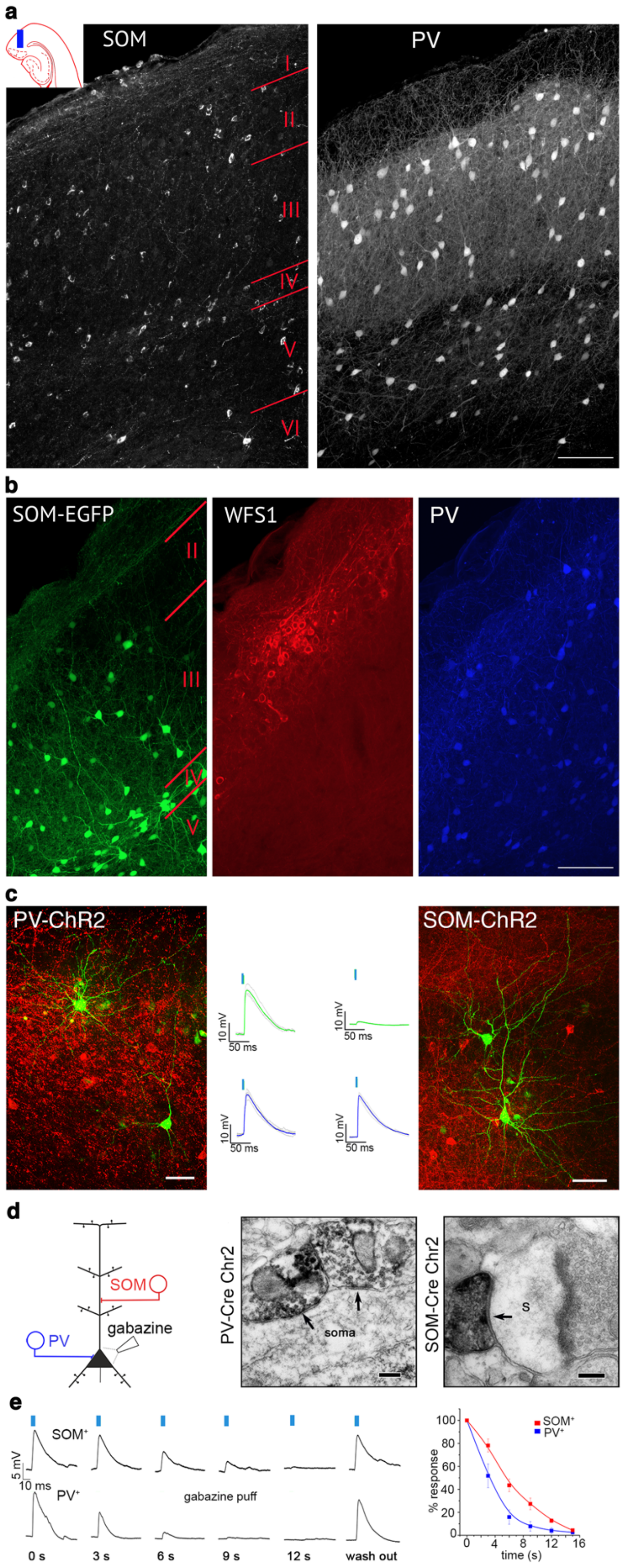

Supplementary Fig. 1. Light-, electron-microscopical and electrophysiological examples showing the different target-selectivity of SOM+ and PV+ interneurons in the MEC. a, inset: schematic drawing of horizontal section of the mice temporal cortex. Blue box represents the imaged area of the dorsomedial MEC. Z-stacked images (30µm total) of SOM (left) and PV (right) immunoreactivity in the MEC. Red lines represent borders between layers. Note that both SOM^+ and PV^+ somata are frequent in $layer_{II\text{-}V}$ and rare or absent in $layer_I$ and $layer_{VI}$. Note that SOM immunoreactivity labels only the somata and some axons, meanwhile PV immunoreactivity labels somata, dendrites and axons. **b**, SOM-Cre-EGFP (green), WFS1 (red) and PV (blue) in the MEC. Red lines represent borders. Note the strong green signal in $layer_I$ due to strong innervation by SOM^+ interneurons. SOM-Cre-EGFP interneurons are heterogenous in size, shape and dendritic morphology. The $WFS1^+$ cell island in $layer_{II}$ is a landmark of dorsal MEC. **c,** Representative examples of recorded neurons in PV-ChR2 (left) and SOM-ChR2 (right) animals. Middle: light induced voltage changes in $layer_{II}$ stellate (green traces) and in $layer_{III}$ pyramidal (blue traces), recorded with CsCl containing intracellular solution. Note the small effect on the stellate cell in SOM-ChR2 animal (right). **d,** Right: Schematic of experimental design recording the kinetics of light evoked events in SOM-ChR2 (red) and PV-ChR2 (blue) animals, after the GABAA receptor blocker gabazine puff on the somatic region of the recorded cell. Left: Electron microscopical images representing the typical targets of $PV\text{-}ChR2^+$ boutons and $SOM\text{-}ChR2^+$ boutons in random samples from $Layer_{I\text{-}V}$ in MEC. **e**, Right: Light evoked responses during gabazine puff at 0, 3, 6, 9, 12 s and wash out in SOM-ChR2 (up) and PV-ChR2 animals. Left: Summarizing plot of postsynaptic responses normalized to the 0 s responses. Note that in PV-ChR2 slices (PV^+, blue) the gabazine puff completely eliminates the postsynaptic PSPs earlier than in SOM^+ slices (SOM^+,

red). Error bars represent standard error of mean (S.E.M.). Scales: a,b: 100 μm, c: 50 μm, d: 200nm.
