# Supplementary material for: Somatostatin expressing GABAergic interneurons in the medial entorhinal cortex preferentially inhibit layerIII-V pyramidal cells
Source: Commun Biol. 2020 Dec 10;3:754. doi: 10.1038/s42003-020-01496-x (PMC7728756; doi:10.1038/s42003-020-01496-x)
Supplement: Supplementary file 3 — Description of Additional Supplementary Files [file 42003_2020_1496_MOESM3_ESM.docx]

Description of Additional Supplementary Files

**File Name:** Supplementary video 1

**Description:**

**Optogenetic activation of the SOM+ GABAergic interneurons in the MEC obstructs short-term memory related behavior.** The animal exploring a Y-maze while the SOM+ interneurons are activated by blue light in the center area (left) or non-exciting red light is irradiated bilaterally to the MEC (right). Note the frequent wrong alternation in the SOM+ interneurons activated (left) and correct alternations in the non-activated (right) session. Both videos are with the same animal. The playback speed of the video has been doubled for better presentation.

**File Name:** Supplementary Data 1

**Description:** Excel table showing all source data for figures.
